# *In vivo* imaging of lung inflammation with neutrophil-specific ^68^Ga nano-radiotracer

**DOI:** 10.1038/s41598-017-12829-y

**Published:** 2017-10-16

**Authors:** Juan Pellico, Ana V. Lechuga-Vieco, Elena Almarza, Andrés Hidalgo, Cristina Mesa-Nuñez, Irene Fernández-Barahona, Juan A. Quintana, Juan Bueren, Jose A. Enríquez, Jesús Ruiz-Cabello, Fernando Herranz

**Affiliations:** 1 Centro Nacional de Investigaciones Cardiovasculares Carlos III (CNIC) and Centro de Investigación Biomédica en Red de Enfermedades Respiratorias (CIBERES). C/Melchor Fernández-Almagro 3, 28029 Madrid, Spain; 2 Division of Hematopoietic Innovative Therapies, Centro de Investigaciones Energéticas Medioambientales y Tecnológicas/Centro de Investigación Biomédica en Red de Enfermedades Raras, 28040 Madrid, Spain; 3 Instituto de Investigación Sanitaria Fundación Jiménez Díaz (CIEMAT/CIBERER/IIS-FJD), 28040 Madrid, Spain; 4 Institute for Cardiovascular Prevention, Ludwig-Maximilians-University, 80336 Munich, Germany; 5 Universidad Complutense de Madrid, 28040 Madrid, Spain

## Abstract

*In vivo* detection and quantification of inflammation is a major goal in molecular imaging. Furthermore, cell-specific detection of inflammation would be a tremendous advantage in the characterization of many diseases. Here, we show how this goal can be achieved through the synergistic combination of nanotechnology and nuclear imaging. One of the most remarkable features of this hybrid approach is the possibility to tailor the pharmacokinetics of the nanomaterial-incorporated biomolecule and radionuclide. A good example of this approach is the covalent binding of a large amount of a neutrophil-specific, hydrophobic peptide on the surface of ^68^Ga core-doped nanoparticles. This new nano-radiotracer has been used for non-invasive *in vivo* detection of acute inflammation with very high *in vivo* labelling efficiency, i.e. a large percentage of labelled neutrophils. Furthermore, we demonstrate that the tracer is neutrophil-specific and yields images of neutrophil recruitment of unprecedented quality. Finally, the nano-radiotracer was successfully detected in chronic inflammation in atherosclerosis-prone ApoE^−/−^ mice after several weeks on a high-fat diet.

## Introduction

Non-invasive quantitative detection of lung inflammation is highly desirable for assessing pathogenic processes in the lung. Inflammatory-cell activation is currently assessed by combining anatomical information obtained by computed tomography with molecular and cellular information obtained from invasive lung biopsy, histopathology and bronchoalveolar lavages^1^. This difficult and time-consuming approach explains the numerous attempts to produce a reliable probe for non-invasive *in vivo* diagnosis^2,3^. Neutrophils are an essential part of the inflammatory cascade, being the first cell type to migrate from the bloodstream to the site of inflammation. One of their most important functions is to eliminate pathogens by engulfing them and liberating enzymes and reactive oxygen species^4,5^. However, neutrophil invasion can cause major tissue damage in acute processes such as lung injury and in chronic diseases such as chronic obstructive pulmonary disease (COPD) and asthma^6^. This multifaceted role underlies interest in designing an effective tool for non-invasive detection, tracking, and quantification of neutrophils. The most promising approach is molecular imaging with a selective tracer, and several probes have been developed to exploit the superb sensitivity of positron emission tomography (PET) and fluorescence tomography or microscopy^1,7–9^.

A particularly interesting compound is the peptide N-cinnamoyl-F-(D)L-F-(D)L-F (cFLFLF), an antagonist of formyl peptide receptor 1 (FPR-1). The very high binding affinity of cFLFLF (K_d_ = 2 nM) is the main reason for its use in neutrophil-specific probes for PET and optical fluorescence^7,8,10^. However, the high hydrophobicity of cFLFLF produces very poor target-to-background ratios^7^. This serious limitation is the reason why to date the amount of radiotracer reaching the inflammation site is below 1% (expressed as the % of injected dose per mass of the organ)^7,10^.

The combined use of nanotechnology and radiochemistry is an attempt to join the best of both approaches: size-dependent properties and unparalleled sensitivity with high *in vivo* selectivity. This combination has been successfully used for many applications, particularly cancer diagnosis^11,12^. Recently, we showed how iron oxide nanoparticles can be core-doped with ^68^Ga for enhanced molecular imaging^13^. Here, we have developed a modified version of these particles, with citrate molecules instead of dextran as the coating. These ^68^Ga-based citrate-coated nano-radiotracers (^68^Ga-NRT) not only provide signal simultaneously in PET and MRI, but, crucially to our approach, also have a very large and highly hydrophilic surface due to the large organic coating per particle^13,14^. We hypothesized that ^68^Ga-NRT particles would overcome the solubility problems encountered with cFLFLF, permitting its use for molecular imaging of neutrophil-driven acute inflammation.

To test our hypothesis, we used a well-characterized model of acute inflammation in mice, based on intratracheal administration of lipopolysaccharide (LPS), which produces neutrophil recruitment in the lungs 24 h post-instillation^15^. PET analysis after intravenous injection of the cFLFLF-functionalized ^68^Ga-NRT enabled *in vivo* detection of neutrophils infiltrating the LPS-treated lungs with very high signal to noise ratios. The ^68^Ga-NRT signal was absent from the lungs of LPS-treated neutrophil-depleted mice, while it was present in LPS-treated macrophage-depleted mice, demonstrating the specificity of the tracer for neutrophils versus macrophages. Finally, we tested the tracer in ApoE^−/−^ mice. Several reports show that after a few weeks on a high-fat diet (HFD) these mice develop lung inflammation featuring invasion by macrophages and neutrophils. Here, we demonstrate that the ^68^Ga-NRT enables detection of this early-stage condition by *in vivo* PET imaging.

## Results

### Synthesis and functionalization of ^68^Ga nano-radiotracers

**Figure 1:**
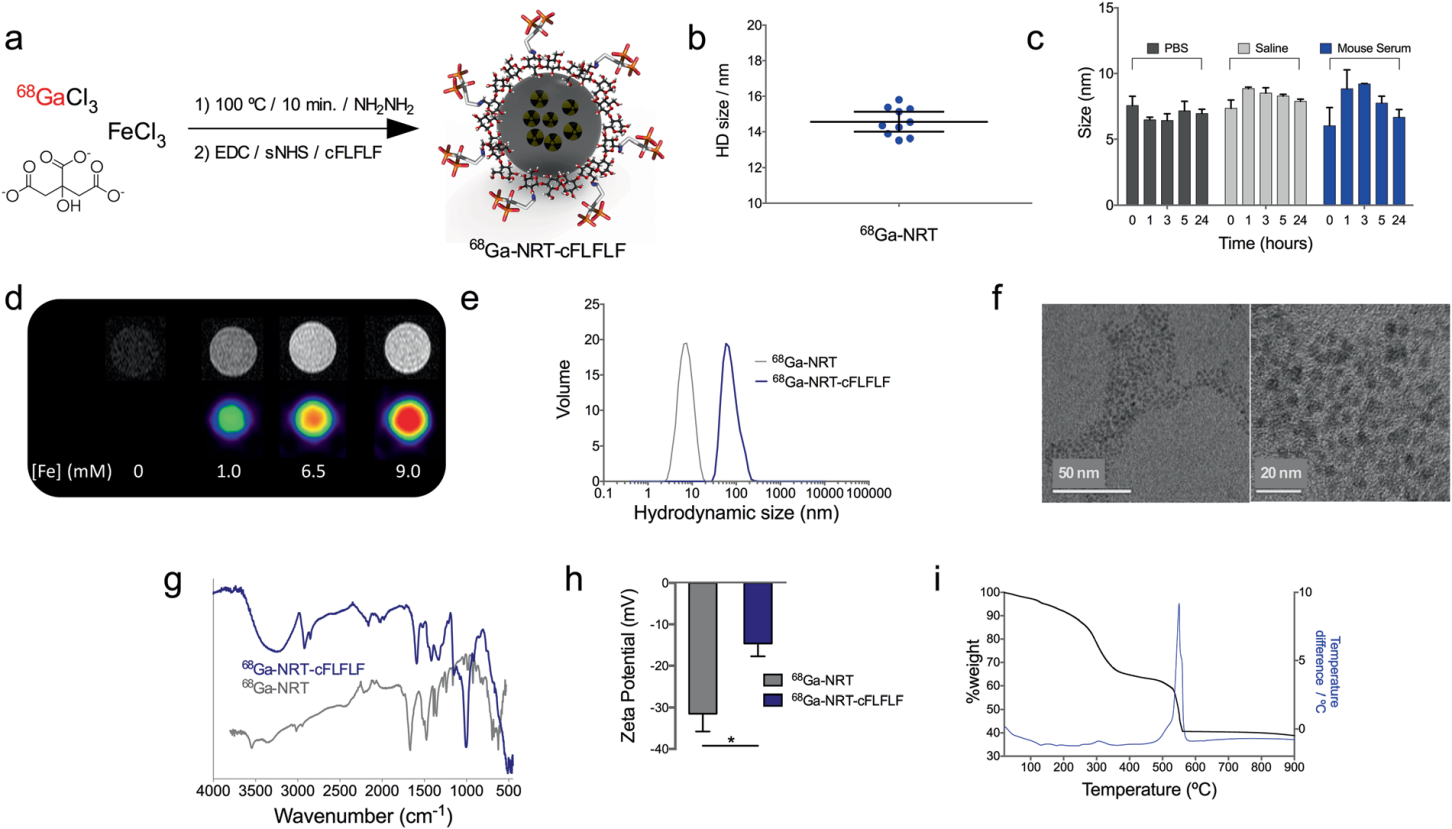
(**a**) Synthesis and functionalization of ^68^Ga core-doped nanoradiotracers. (**b**) Hydrodynamic size (Z-average), determined by dynamic light scattering, of ten independent batches of citrate-coated NRT (blue dots); horizontal bars represent mean and 95%CI. (**c**) Hydrodynamic size (maximum peak in volume) of citrate-coated NRT in PBS, saline and mouse serum (from t = 0 to 24 hours). (**d**) PET images for different gallium concentrations in the ^68^Ga-NRT. (**e**) ^68^Ga-NRT hydrodynamic size, measured by dynamic light scattering, before and after conjugation of cFLFLF peptide. (**f**) TEM images of ^68^Ga-NRT-cFLFLF at two magnifications (scale bars are 50 nm and 20 nm). (**g**) Comparison of FTIR spectra before and after conjugation of cFLFLF peptide. (**h**) Zeta potential before and after cFLFLF conjugation (statistical analysis by two-tailed *t*-test; error bars indicate s.d., N = 3; *, *t* 4.698 *P* 0.0182). (**i**) Thermogravimetric curve of ^68^Ga-NRT-cFLFLF.

We recently reported the use of ^68^Ga core-doped nanoparticles as a novel platform for molecular imaging^13^. Here, we have used citrate instead of dextran as the coating agent. Briefly, a mixture of FeCl_3_, sodium citrate, ^68^GaCl_3_ (eluted from a ^68^Ge/^68^Ga generator), and hydrazine was heated at 100 °C in a synthesis microwave oven (Fig. 1a). After purification by gel chromatography, we obtained hydrophilic and extremely small nano-radiotracers, 2.7 ± 1.0 nm of core size measured by STEM-HAADF (Fig. S1) and 14.5 ± 2.1 nm hydrodynamic size (HD). The yield of the synthesis was 26.0% Fe and a 92% radiolabelling, for a final specific activity of 7.1 GBq/mmol Fe. Ten independent repetitions of the synthesis on different days demonstrated excellent HD reproducibility (Fig. 1b). The particles show a very thick organic coating, 47% weight measured by thermogravimetry (Fig. S2). The small size and very thick organic layer of these NRT favour excellent colloidal stability. This property is essential for our goal to conjugate a highly hydrophobic peptide, which under other conditions would be very difficult to stabilize. Colloidal stability was demonstrated by the maintenance of hydrodynamic size and the absence of NRT aggregation up to 24 hours after dispersion in PBS, saline and mouse serum (Fig. 1c). Testing of the PET and MRI signals in ^68^Ga-NRT phantoms demonstrated successful incorporation of the radioisotope, with the signal increasing in proportion with the amount of NRT (Fig. 1d). As we have already demonstrated, with this synthetic protocol and purification, this is an indication of core-doping with the radioisotope^13^. Positive signal in MRI phantom is in agreement with the data measured by relaxometry for *r*_1_ and *r*_2_, 6.8 mM^−1^s-1 and 15.9 mM^−1^s^−1^ respectively.

After characterizing ^68^Ga-NRT performance, we covalently attached the neutrophil-specific peptide cFLFLF to citrate carboxyl groups by the classical EDC/Sulfo-NHS reaction, yielding ^68^Ga-NRT-cFLFLF. Conjugation of cFLFLF increased NRT hydrodynamic size from 14.5 nm to 83.3 ± 10.5 nm (Fig. 1e). Despite the increased hydrodynamic size, we did not observe aggregation upon storage or in the *in vivo* experiments; TEM images show a core size of 2.5 ± 0.5 nm, also with no sign of aggregation (Fig. 1f). This increase in hydrodynamic size, but not core size, can therefore be attributed to the large number of highly hydrophobic peptides attached to the NRT surface. Successful peptide attachment was confirmed by the presence of bands on FTIR at 2913 cm^−1^ (aromatic C-H), 996 cm^−1^ (olefin) and 770 cm^−1^ (benzene ring) (Fig. 1g). Further evidence of peptide attachment is provided by the zeta potential, which changed from a mean value of −31.5 mV for ^68^Ga-NRT to −14.6 mV for ^68^Ga-NRT-cFLFLF, due to the transformation of some of the negatively charged carboxylate groups into amides (Fig. 1h). The thermogravimetric curve shows the typical profile for this type of nanoparticles, with a very small core and a thick organic coating. At 560 °C, a strong exothermic process further indicates the presence of the peptide, accounting the very high figure of 23% of total NRT mass, corresponding to 1 mmol of peptide per 90 mmol Fe.

### Cytotoxicity analysis of ^68^Ga-NRT-cFLFLF in primary hematopoietic progenitor cells and mature neutrophils

The potential cytotoxic effects of ^68^Ga-NRT-cFLFLF were studied by *in vitro* colony forming cell (CFC) assay in primary hematopoietic progenitor cells, as highly-proliferative primary cells are particularly sensitive to toxic compounds. To induce the formation of myeloid and erythroid colonies, we cultured freshly isolated cord blood CD34^+^ cells from healthy donors in methylcellulose medium in the presence of cytokines. These assays were conducted in the absence or presence of different concentrations of ^68^Ga-NRT-cFLFLF. The NRT concentrations used in the CFC assays were up to 50 times the expected *in vivo*-infused NRT dose; 3 μg of Fe, 15 μg of Fe and 150 μg of Fe.

**Figure 2:**
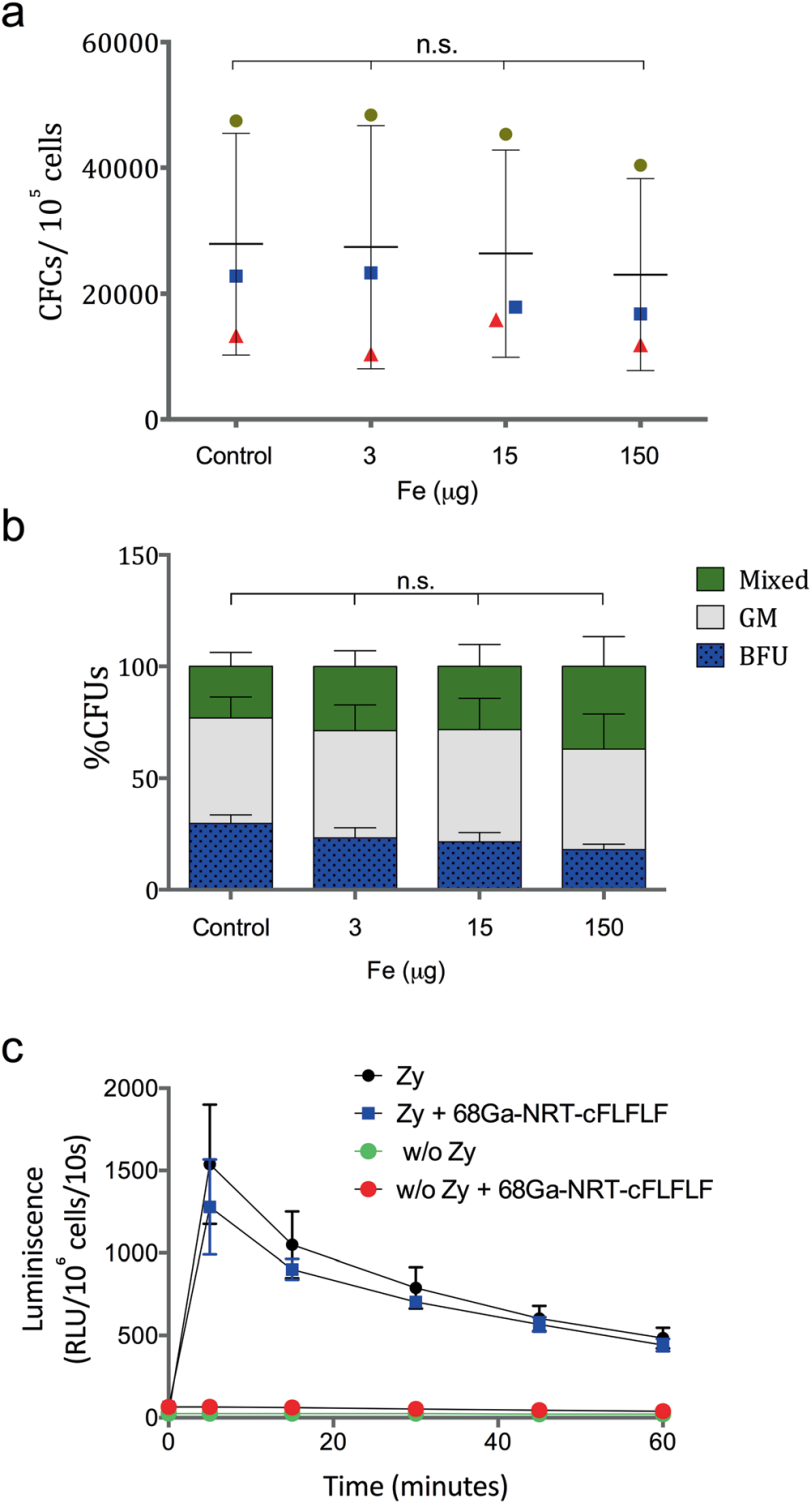
(**a**) Analysis of hematopoietic colonies generated in the absence and the presence of different concentrations of ^68^Ga-NRT-cFLFLF (total number of colonies in 10^5^ human cord blood cells), symbol colours and shape indicate cord number. (**b**) Distribution of different colony types in CFU assays. The proportions of BFU-E, CFU-GM, CFU-GEMM are shown. (**c**) Analysis of the respiratory burst *in vitro* CD34^+^-derived neutrophils. The respiratory burst response was induced with C3bi-opsonized zymosan (zy) and detected by luminol-enhanced chemiluminescence. Luminescence detected for each sample was extrapolated for 10^6^ cells with the following equation: 10^6^ cell luminescence = sample luminescence ∗ 10^6^ cells/cell number.

The total number of colonies generated at any ^68^Ga-NRT-cFLFLF dose was similar to the number generated in unexposed cultures (Fig. 2a), indicating that ^68^Ga-NRT-cFLFLF is not toxic to human hematopoietic progenitor cells even at the highest concentration used. Data show the different values per cord. Values show a large standard deviation, which is expected in cord blood samples^16^. However, it is clear the lack of effect due to the NRT in each cord. Statistical analysis by ANOVA rendered no significant differences between control and experimental groups (*P* > 0.05, N = 3). In the CFC assays, we distinguished three types of colonies: erythroid burst-forming units (BFU-E), granulocyte-monocyte colony-forming units (CFU-GM), and granulocyte, erythrocyte, monocyte, megakaryocyte colony-forming units (CFU-GEMM). The colony-type profile was not affected by NRT exposure, additionally indicating that lineage commitment and differentiation were unaffected by ^68^Ga-NRT-cFLFLF. However, we have to take into account that at the highest concentration of ^68^Ga-NRT-cFLFLF, due to the presence of high concentrations of iron, it is difficult to distinguish red coloured erythroid colonies.

Since the cFLFLF peptide targets the formyl peptide receptor-1 (FPR-1) on neutrophils, it was important to determine whether binding of ^68^Ga-NRT-cFLFLF altered neutrophil function, in particular the capacity for the oxidative burst in response to pathogens. We therefore differentiated freshly isolated CD34+ cells into neutrophils *in vitro*. These neutrophils were then subjected to a chemiluminescence oxidative burst assay (Fig. 2b). Also in this case, statistical analysis by ANOVA rendered no significant differences between control and experimental groups (*P* > 0.05, N = 3). Treatment with ^68^Ga-NRT-cFLFLF in the absence of zymosan stimulation induced no response in *in vitro* differentiated neutrophils, indicating that the tracer by itself is unable to stimulate the oxidative burst. In response to zymosan, the oxidative burst was similar in the absence and presence of ^68^Ga-NRT-cFLFLF (*P* > 0.05, Mann-Whitney non parametric test), indicating that the presence of the tracer does not alter cell function (Fig. 2c).

### Inflammation model in C57BL/6 mice

The endotoxin lipopolysaccharide (LPS) is commonly used to experimentally induce acute lung inflammation. In this model, an acute transient inflammation elicits maximum neutrophil recruitment 24 hours after intratracheal LPS administration^17,18^. This approach has been used to model conditions such as chronic obstructive pulmonary disease (COPD) and asthma^19^. LPS (50 µg) was administered by intratracheal instillation to 12-week-old C57BL/6 mice, followed by perfusion and excision of the lungs at 24 h post treatment. Neutrophil accumulation was assessed by flow cytometry of the perfusate. The lungs of LPS-instilled mice had a markedly higher neutrophil content than control mice instilled with PBS, demonstrating LPS-induced neutrophil recruitment (Fig. S3). Histological analysis of lung sections confirmed hyaline membrane formation and neutrophil infiltration, a typical feature of LPS-induced lung injury (Fig. S3).

### *In vivo* detection of neutrophils by PET in LPS-treated model

**Figure 3:**
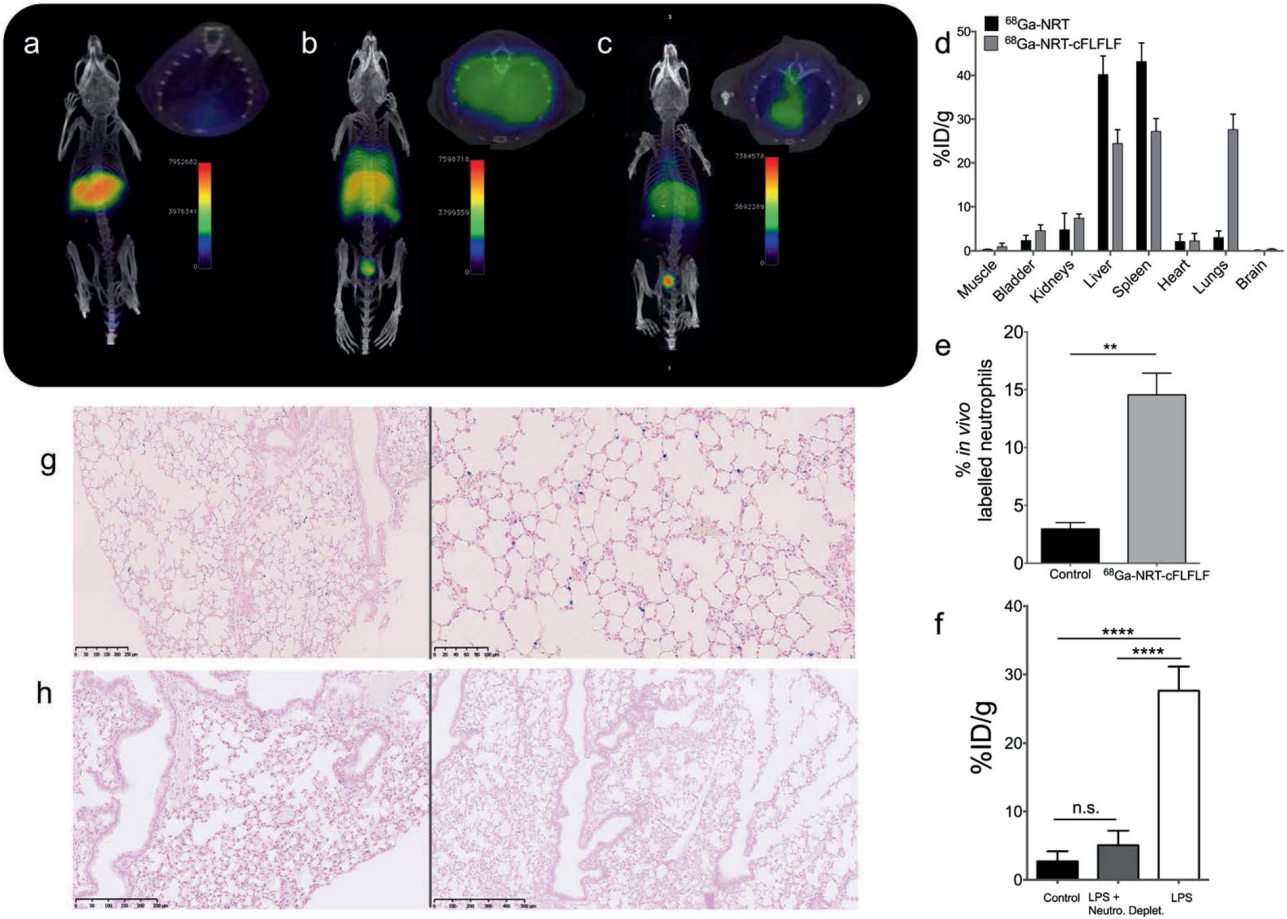
(**a**) PET/CT imaging of a C57BL/6 mouse, without LPS instillation, 1 h post *i.v*. injection with ^68^Ga-NRT-cFLFLF. (**b**) PET/CT imaging of a mouse with LPS-induced pulmonary inflammation (50 µg, tracheal instillation) 1 h post *i.v*. injection with ^68^Ga-NRT-cFLFLF. (**c**) PET/CT image of a neutrophil-depleted LPS-treated mouse 1 h post *i.v*. injection with ^68^Ga-NRT-cFLFLF. (**d**) Radiotracer organ biodistribution expressed as the percentage injected dose per gram (%ID/g) in LPS-instilled mice injected with ^68^Ga-NRT or ^68^Ga-NRT-cFLFLF (N = 5). (**e**) Percentage of *in vivo* labelled neutrophils measured by flow cytometry in LPS-instilled mice without nanoparticle injection (control) and 1 h post *i.v*. injection with fluorophore-conjugated ^68^Ga-NRT-cFLFLF. **, *t* 5.911, *P* 0.0038 by two-tailed *t*-test; error bars indicate s.d., N = 5, experiment repeated twice. (**f**) Radiotracer accumulation (%ID/g) in the lungs of LPS-instilled mice injected with ^68^Ga-NRT (black) or ^68^Ga-NRT-cFLFLF (white) and in the lungs of neutrophil-depleted LPS-instilled mice injected with ^68^Ga-NRT-cFLFLF (dark grey). ****P < 0.0001, n.s. *P* > 0.05, one-way ANOVA; error bars indicate s.d., N = 5, experiment repeated thrice. (**g**) Prussian blue staining of lung sections from an LPS-instilled mouse injected with ^68^Ga-NRT-cFLFLF (scale bars are 250 µm and 100 µm). (**h**) Prussian blue staining of lung sections from a neutrophil-depleted LPS-instilled mouse injected with ^68^Ga-NRT-cFLFLF (scale bars are 250 µm and 500 µm).

The efficiency of ^68^Ga-NRT-cFLFLF as a neutrophil-specific radiotracer was assessed *in vivo* PET/CT imaging experiments (Fig. 3). We first conducted two control analyses. In one, ^68^Ga-NRT-cFLFLF particles were administered intravenously to healthy C57BL/6 mice, and images were acquired one hour post-injection. No ^68^Ga signal was observed in the lungs (Fig. 3a), whereas strong signals appeared in the liver and spleen. This biodistribution is expected for nanoparticles with this size and coating, and demonstrates that the particles do not accumulate passively in the target organ (lung) due to an effect of hydrodynamic size or through non-specific cellular recognition of the peptide. In the second control, three LPS-treated mice received systemic injections of ^68^Ga-NRT with no peptide attached (Fig. S4). This NRT control allowed us to demonstrate that the particles do not passively accumulate in the lungs of LPS-treated mice. As we expected, the precursor nanoparticles were removed from the circulation through the normal clearance organs, mainly liver and bladder, due to their small hydrodynamic size, at the limit between renal filtration and hepatic elimination. With these controls completed, we proceeded to the study of ^68^Ga-NRT-cFLFLF in mice (N = 5) with LPS-induced lung inflammation. The results show a clear uptake of ^68^Ga-NRT-cFLFLF in the lungs together with accumulation in the major reticuloendothelial system organs (Fig. 3b).

The biodistribution data is quite revealing, in agreement with imaging results, showing a very large accumulation in the lungs when ^68^Ga-NRT-cFLFLF is used in the pulmonary inflammation model, with a percentage injected dose per gram (%ID/g) of almost 30% compared to 3% in the controls (Fig. 3d). Furthermore, in a different experiment with the same experimental protocol, neutrophils in the lungs were isolated 1 hour post i.v. injection of dye-labeled ^68^Ga-NRT-cFLFLF and their labelling calculated mounting for and outstanding 15% of *in vivo* labelled neutrophils, compared to less than 3% in control experiment (Fig. 3e).

The results up to this point seemed to confirm that our NRT was able to detect the inflammatory process after LPS treatment and that, in this detection, the *in vivo* labelling of neutrophils was a major contributor. However, to demonstrate selectivity towards neutrophils two further experiments were designed. It is well known the avidity of macrophages for nanosized particles and the important role and accumulation of these cells in inflammatory processes and imaging^20^. Thus, it could be argued that the increased signal intensity in the lungs could be due to pronounced presence of macrophages capturing the NRT after the adsorption of opsonins in systemic circulation, and as result they could not show specificity towards neutrophils. Supporting this contention, some studies have also shown some affinity towards macrophages by the cFLFLF peptide^21,22^. To check this option, first we performed an additional experiment in which LPS-instilled mice were further treated with Ly6G antibody for neutrophils depletion^23^. In this case, without the transient presence of neutrophils, the specific uptake of the neutrophilic targeted nanoprobe in the lungs should be much lower or, ideally, disappear. The complete disappearance of signal uptake in the lungs after neutrophilic depletion in acute inflammatory animals (Fig. 3c) demonstrated the specificity of nanotracers comprising a formyl peptide receptor binding moiety toward this type of leukocytes. Moreover, we found that, after neutrophil depletion, the NRT circulate for longer times in the bloodstream with a clear signal in the heart not displayed previously (Fig. 3c, axial view).

**Figure 4:**
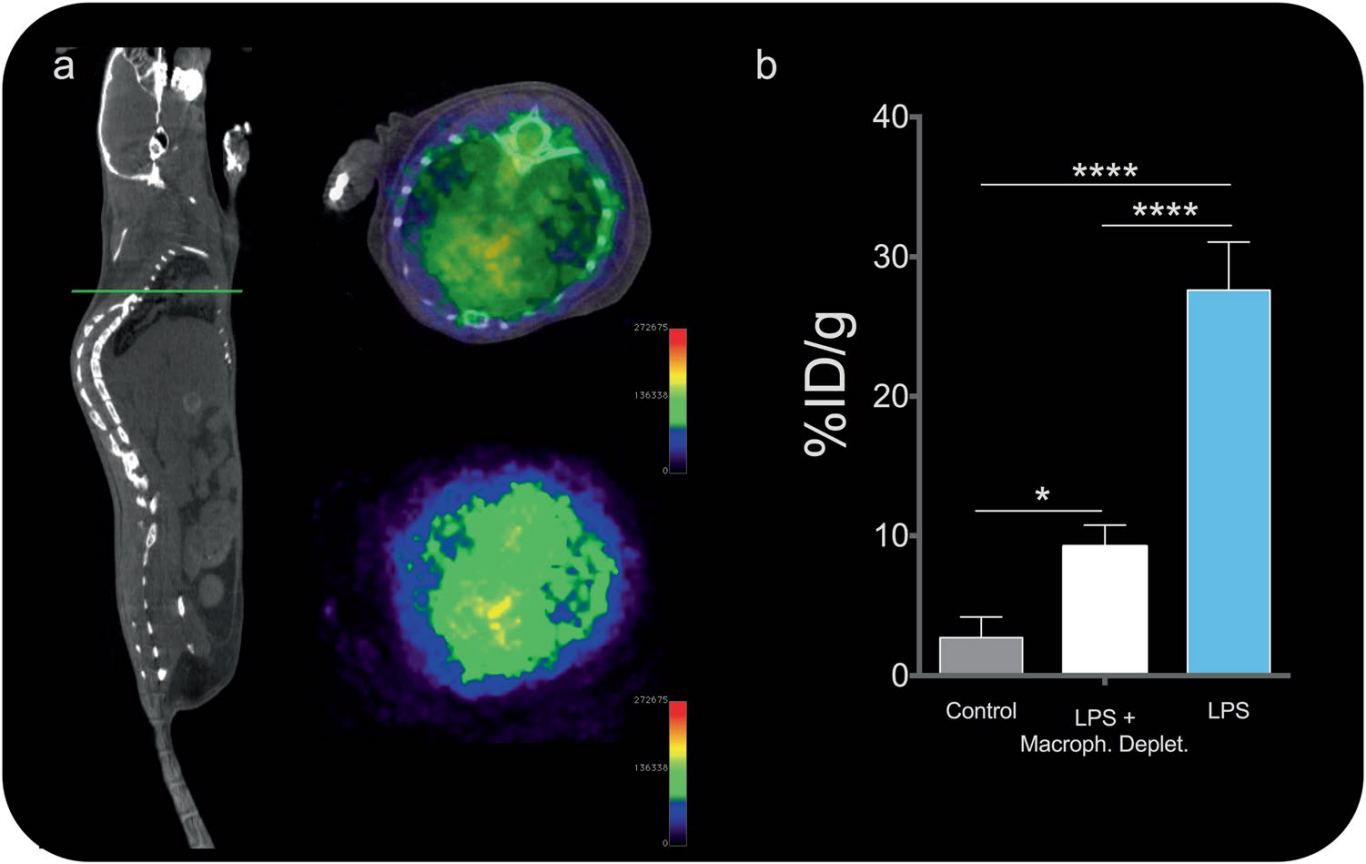
(**a**) PET/CT image of a macrophage-depleted LPS-treated mouse 1 h post i.v. injection with ^68^Ga-NRT-cFLFL F. Left, whole body CT image of a representative mouse, green line indicates the plane which PET/CT and PET is shown next. (**b**) Radiotracer accumulation (%ID/g) in the lungs of LPS-instilled mice injected with ^68^Ga-NRT (black) or ^68^Ga-NRT-cFLFLF (white) and in the lungs of macrophage-depleted LPS-instilled mice injected with ^68^Ga-NRT-cFLFLF (grey). ****P < 0.0001, *P < 0.05, one-way ANOVA; error bars indicate s.d., N = 3. Experiment repeated twice.

After imaging we measured radiotracer accumulation in isolated organs was measured *ex vivo* with a gamma counter (Fig. 3f). In agreement with the imaging results, radiotracer accumulation in the lungs revealed a very high accumulation of ^68^Ga-NRT-cFLFLF in inflamed lungs, with a 27.6%ID/g in the lungs of LPS-instilled mice compared with 5%ID/g in neutrophil-depleted mice and 3%ID/g in mice injected with non-peptide-conjugated ^68^Ga-NRT (Fig. 3f). Consistent with the *in vivo* and gamma counter results, Prussian blue staining on histological sections after decay of the radioactive signal revealed iron-containing ^68^Ga-NRT-cFLFLF particles (blue dots) in the lungs of mice with LPS-induced lung inflammation (Figs 3g and S5) but not those of similarly treated mice depleted of neutrophils (Figs 3h and S5) or of mice without induced neutrophil recruitment (Fig. S5). After this, we performed a second experiment in which LPS-instilled mice were previously treated with clodronate, for macrophage depletion^23^. This treatment transiently depletes macrophages but, importantly, neutrophil recruitment to the site of infection is strongly reduced^24–26^. In this model and with a NRT specific for neutrophils, we predicted ^68^Ga-NRT-cFLFLF accumulation in the lungs after LPS treatment, but with a reduced uptake compared with macrophage-retaining mice, since the amount of neutrophils reaching the area of inflammation should be lower. The results confirmed our prediction; images (Fig. 4a) and quantification (Fig. 4b) of the uptake in the lungs of macrophages-depleted mice after LPS show robust uptake compared to control mice, but much lower than mice with intact macrophages, in agreement with our hypothesis^26^.

### PET detection of neutrophils during chronic inflammation *in vivo*

**Figure 5:**
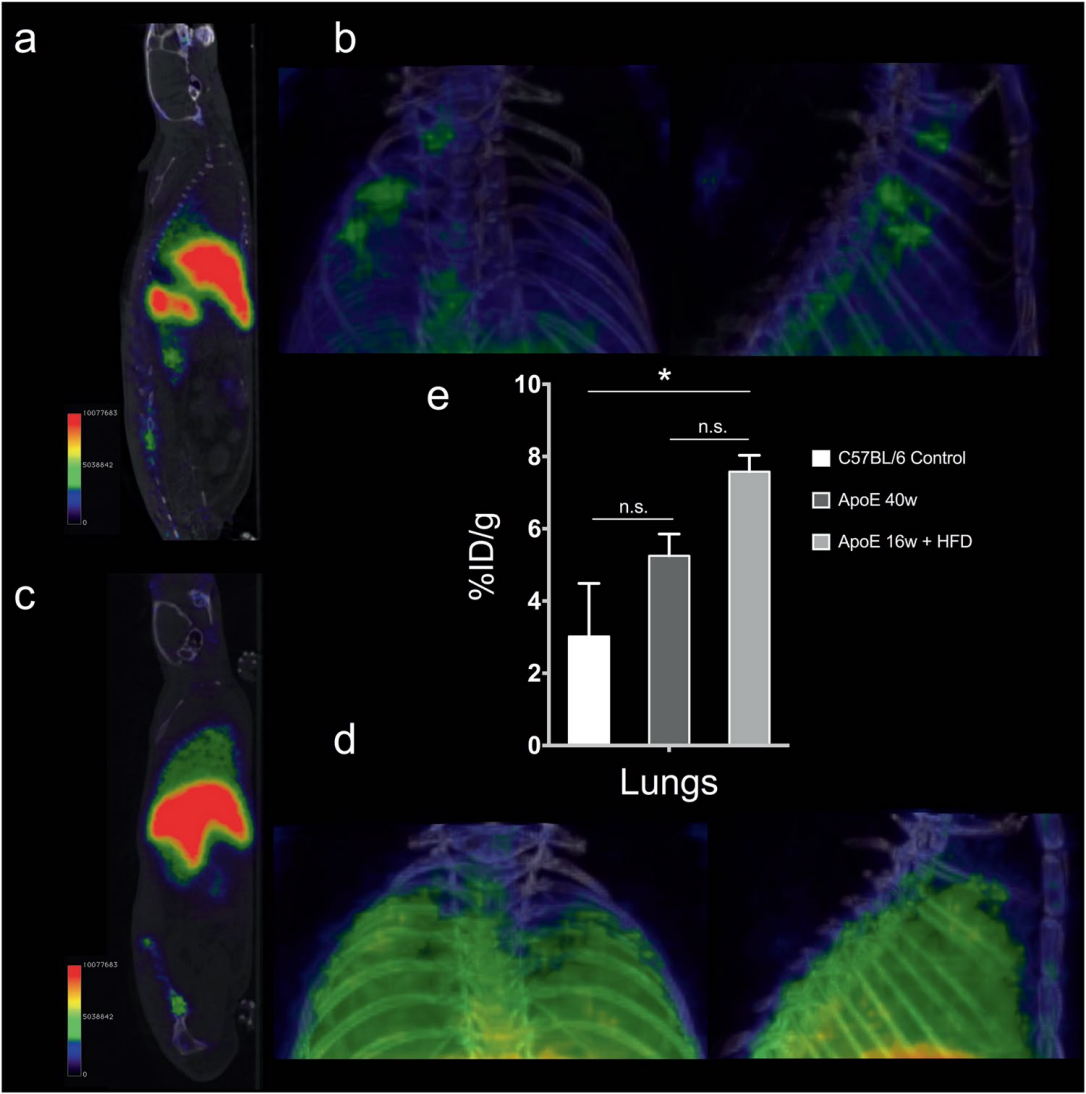
(**a**) PET/CT imaging 1 h post i.v. injection of ^68^Ga-NRT-cFLFLF in 40-week-old ApoE^−^/^−^ mice. (**b**) PET/CT maximum intensity projection of the pulmonary region 1 h post i.v. injection of ^68^Ga-NRT-cFLFLF in 40-week-old ApoE^−^/^−^ mice. (**c**) PET/CT imaging 1 h post i.v. injection of ^68^Ga-NRT-cFLFLF in 16-week-old ApoE^−^/^−^ mice fed a high-fat diet (HFD). (**d**) PET/CT maximum intensity projection of pulmonary region 1 h post *i.v*. injection of ^68^Ga-NRT-cFLFLF in 16-week-old ApoE^−^/^−^ mice fed a high-fat diet (HFD). (**e**) Organ biodistribution expressed as %ID/g in C57BL/6 mice, 40-week-old ApoE^−^/^−^ mice, and 16-week-old HFD ApoE^−^/^−^ mice. *P < 0.05, one-way ANOVA; error bars indicate s.d., N = 3.

After demonstrating the ability of ^68^Ga-NRT-cFLFLF to non-invasively detect neutrophils during acute inflammation, we wanted to test its performance in a mouse model of mild chronic inflammation. Previous studies have shown neutrophilia and monocytosis in ApoE^−/−^ mice fed a high-fat diet (HFD)^27,28^. Moreover, this obesogenic diet induces lung remodelling in ApoE^*−/−*^ mice, featuring recruitment of monocytes and a small number of neutrophils to the lungs^29^. We carried out imaging experiments in aged ApoE^*−/−*^ mice (40 weeks old) fed a standard chow diet and in young ApoE^*−/−*^ mice (16 weeks old) fed the HFD and injected with ^68^Ga-NRT-cFLFLF. It has been shown that the older mice would have higher levels of neutrophils than C57BL/6 mice but without any marked alteration in the lungs, whereas the HFD-fed mice would only show modest recruitment of neutrophils to the lungs. Accordingly, the 40-week-old ApoE^*−/−*^ mice showed a similar biodistribution to controls, with no significant signal in the lungs (Fig. 5a,b). In contrast, the 16-week-old HFD-fed mice showed a strong lung signal (Fig. 5c,d). *Ex vivo* quantification matched the imaging results, showing a higher overall neutrophil content in ApoE^*−/−*^ mice than in controls and a clear increase in the lungs after feeding a high-fat diet.

## Discussion

Inflammation is a central feature of many clinical conditions and can be broadly classified as acute or chronic. Acute inflammation typically occurs over a timescale of minutes to hours, whereas chronic inflammation can develop over several days to months. The predominant cell type in acute inflammation is the neutrophil, whereas chronic inflammation features a greater variety of cell types. The key role played by inflammation in many diseases places a premium on the development of methods for its *in vivo* diagnosis, especially methods with specificity towards a particular receptor up-regulated by inflammatory stimuli or for the accumulation of different cell types in the area under study^30^. The interest in neutrophil identification has attracted attention to cFLFLF for the development of molecular imaging probes. This formyl peptide receptor-1 antagonist shows selectivity towards neutrophils with very high binding affinity (K_d_ = 2 nM). However, its use has been limited due to its hydrophobic nature, which has produced poor signals and very low *in vivo* labelling. We reasoned that this problem could be overcome through the incorporation of cFLFLF into highly hydrophilic nanoplatforms and their detection by nuclear imaging. We used a new generation of nano-radiomaterials in which the radioisotope is incorporated in the nanoparticle core by very fast temperature ramping in a synthesis microwave. This resulted in very high peptide incorporation in the NRT (1 mmol per 90 mmol Fe by TG analysis and FTIR spectroscopy) and a reduction of the zeta potential from −31.5 mV to −14.6 mV. Even with the mild increase hydrodynamic size due to the incorporated peptide, these physiochemical features ensure NRT stability and its successful use *in vivo*. This is due not only to the large amount of peptide incorporated per nanoparticle, but also and more importantly, to the good value obtained for the specific activity (700 GBq/mmol peptide). The utility of this approach is demonstrated in a model of acute inflammation in the lung, in which the NRT particles selectively accumulate in the lungs of animals treated with LPS, producing very clear images and labelling about 15% of neutrophils *in vivo*, the highest reported value for this type of approach. This high labelling permits the unambiguous non-invasive identification of acute inflammation in the lungs. Furthermore, the ^68^Ga-NRT-cFLFLF tracer shows high selectivity towards neutrophils. Indeed, cFLFLF is highly neutrophil selective; however, some reports also show interaction with macrophages. To check the situation in our model, we depleted LPS-administered mice of neutrophils. Interestingly, the neutrophil depletion method requires the presence of macrophages. Imaging confirmed the loss of the LPS-dependent ^68^Ga-NRT-cFLFLF signal in the lungs of neutrophil-depleted mice. Furthermore, in a macrophage-depleted model the signal in the lungs was still statistically significant compared to control mice and images showed clear accumulation, this is particularly remarkable since neutrophil recruitment is hampered in this model.

The utility of ^68^Ga-NRT-cFLFLF for the detection of chronic inflammation was evaluated in ApoE^*−/−*^ mice, which show a mild recruitment of neutrophils that is exacerbated in the lungs upon feeding a high-fat diet for several weeks. Even in the setting of this less pronounced lung remodelling and neutrophil recruitment, the ^68^Ga-NRT-cFLFLF radiotracer revealed clear differences between aged and young HFD-fed ApoE^*−/−*^ mice and between aged ApoE^*−/−*^ mice fed the HFD and those fed a normal diet.

The findings presented here demonstrate how nanotechnology and nuclear imaging can be combined to overcome the limitations of traditional approaches, yielding a new tool for the non-invasive detection of inflammation with *in vivo* selectivity towards neutrophils. These results pave the way to the non-invasive identification of neutrophils in a number of highly relevant inflammatory disorders.

## Methods

^68^Ga (t½ = 68 min, β += 89% and EC = 11%) was obtained from a ^68^Ge/^68^Ga generator system (ITG Isotope Technologies Garching GmbH, Germany) in which ^68^Ge (T½ = 270 d) was attached to a column based on organic matrix generator. The ^68^Ga was eluted with 4 mL of 0.05 M hydrochloric acid. Iron (III) chloride, hydrazine monohydrate, N-(3-Dimethylaminopropyl)-N′-ethylcarbodiimide hydrochloride and N-hydroxysulfosuccinimide sodium salt were purchased from Sigma-Aldrich. Citric acid trisodium salt dihydrate was purchased from Acros organics. cFLFLF peptide was purchased from Biomedal.

### Synthesis of ^68^Ga-NRT

FeCl_3_ × 6 H_2_O (75 mg, 0.28 mmol), sodium citrate hydrate (80 mg, 0.27 mmol) and 1280 MBq of ^68^GaCl_3_ in HCl (0.05 M, 4 mL) were dissolved in water (5 mL) in a microwave-adapted flask, followed by addition of 1 mL hydrazine hydrate. The solution was ramped to 100 °C over 54 s and held at this temperature for 10 minutes (240 W) in a Monowave 300 microwave reactor equipped with an internal temperature probe and an external IR probe (Anton Paar, GmbH73760, Ostfildern-Scharnhausen, Germany). The reaction mixture was then cooled to 60 °C and the ^68^Ga-NRT producte was purified by passing the mixture through a PD-10 column to eliminate excess small reagents, including all unincorporated radiotracer. This purification process provided 9 mL of ^68^Ga-NRT with a total activity of 781 MBq (measured 40 minutes after starting the reaction), a radiolabelling yield of 92%.

### Synthesis of ^68^Ga-NRT-cFLFLF

To 770 MBq of ^68^Ga-NRT (2.25 mL) were added 0.07 mmol of N-(3-dimethylaminopropyl)-N′-ethylcarbodiimide hydrochloride (EDC) and 0.075 mmol of N-hydroxysulfosuccinimide sodium salt (Sulfo-NHS). The solution was stirred for 30 min at room temperature (r.t.) and then ultracentrifuged at 10,350 x g through Amicon 30 kDa centrifugal filters for 4 min to remove excess reagents. The retentate was resuspended in 1.5 mL PBS, pH 8, and 1 mg of cFLFLF dissolved in 50 µL DMSO was added to the solution. The mixture was maintained at r.t for 60 min with stirring. Finally, another ultrafiltration step was performed to eliminate unreacted peptide. The retentate was resuspended in saline solution, yielding 218.9 MBq of ^68^Ga-NRT-cFLFLF, a radiolabelling yield of 97%.

### Animal model

Mice were housed in the specific pathogen-free facilities at the Centro Nacional de Investigaciones Cardiovasculares, Madrid. All animal experiments conformed to EU Directive 2010/63EU and Recommendation 2007/526/EC, enforced in Spanish law under Real Decreto 53/2013PET imaging. Protocol approved by Madrid regional government (PROEX16/277).

Inflammation model was obtained by intratracheal instillation of LPS (50 µg) to 8–12-week-old C57BL/6 mice, followed by perfusion and excision of the lungs at 24 h post treatment.

### MRI relaxation properties of NRT samples

Longitudinal and transverse relaxation times were measured for four concentrations of each nanoparticle sample in a Bruker Minispec mq60 contrast agent analyzer at 1.5 T and 37 °C. R_1_ and R_2_ values were plotted against the Fe mM concentration (0, 0.25, 0.5, 1, 2).

*In vivo* PET/CT imaging in mice was performed with a nanoPET/CT small-animal imaging system (Mediso Medical Imaging Systems, Budapest, Hungary). List-mode PET data acquisition commenced 1 hour after injection of a bolus of 10 MBq of ^68^Ga-NRT-cFLFLF through the tail vein and continued for 30 minutes. At the end of PET, microCT was performed for attenuation correction and anatomic reference. The dynamic PET images in a 105 × 105 matrix (frame rates: 3 × 10 min, 1 × 30 min, 1 × 60 min) were reconstructed using a Tera-Tomo 3D iterative algorithm. Images were acquired and reconstructed with proprietary Nucline software (Mediso, Budapest, Hungary). Images were analyzed using Osirix software (Pixmeo, Switzerland).

### *Ex vivo* biodistribution

Biodistribution was studied with a Wizard 1470 gammacounter (Perkin Elmer). Animals were sacrificed in a CO_2_ chamber, after which blood was extracted and the animals perfused with 8 mL PBS. Organs were extracted and counted in the gammacounter for 1 min each. Readings were decay corrected presented as the percentage injected dose per gram (%ID/g).

### Purification of cord blood CD34^+^ cells

Cord Blood samples from healthy donors were obtained from the Madrid Community Transfusion Centre. Mononuclear cells were purified by density gradient centrifugation in Ficoll-Paque PLUS medium (GE Healthcare, Fairfield, USA). CD34^+^ cells were selected using the CD34 MicroBead Kit. Magnetic-labelled cells were positively selected first with an LS column in a QuadroMACS™ separator and then with an MS column in an OctoMACS™ separator (all from MACS, Miltenyi Biotec, Bergisch Gladbach, Germany). FACS analysis routinely revealed a CD34^+^ purity of 80–95%.

### Progenitor assays in culture

Colony forming cell (CFU) assays were performed by adding 250 cells from each condition to 1 ml of methylcellulose-based medium (#130-091-280 MACS, Miltenyi Biotec) in the presence or absence of nanoparticles at varying concentrations. Cultures were plated in triplicate in 35 mm dishes (#430165, Corning) and grown at 37 °C, 5% CO_2_. After culture for 14 days, colonies were observed under an inverted microscope (Nikon Phase Contrast ELWD 0.3 133909, Japan) and classified and counted according to precursor type: erythroid burst-forming units (BFU-E), granulocyte-monocyte colony-forming units (CFU-GM), and granulocyte, erythrocyte, monocyte, megakaryocyte colony-forming units (CFU-GEMM).

### Chemiluminescence assay of the neutrophil respiratory burst

The respiratory burst activity was analyzed by chemiluminescence assay in 12-day *in vitro* differentiated neutrophils exposed to complement-opsonized zymosan, which activates the respiratory burst through CD11/CD18^31,32^. Differentiated neutrophils were obtained by culturing freshly isolated CD34^+^ CB cells for 12 days in neutrophil differentiation medium (IMDM supplemented with 20% Hyclone, hIL-3 20 ng/ml), hSCF (20 ng/ml), hG-CSF (Neulasta, 100 ng/ml), and 1% penicillin/streptomycin). The luminol-enhanced chemiluminescence assay was as previously described^33^. Cells (10^5^) were preincubated with 15 μg/mL human serum albumin for 15 minutes in 160 µL RPMI 1640 medium + GlutaMAX in an Isoplate-96 white-frame clear-well microplate (PerkinElmer, Waltham, USA). At the beginning of the assay, 10 μmol/L luminol (Sigma-Aldrich, St. Louis, USA) and 1 mg/mLopsonized zymosan were added to the reaction mixture. Luminol-enhanced chemiluminescence was read at 10-s intervals at the designated time points with a Genios Pro reader (Tecan, Männedorf, Switzerland). The assay was performed at room temperature and chemiluminescence was reported as relative light units (RLU)/10^6^ cells/10 s.

Zymosan (Sigma-Aldrich, St. Louis, USA) was opsonized with human serum as previously described^34^. Briefly, zymosan was resuspended in PBS at 20 mg/mL, heated and shaken at 100 °C for 20 minutes, sonicated for 60 s, and washed in PBS by centrifugation (300 g, 2 min). Finally, Zymosan was resuspended in fresh PBS at 20 mg/mL and incubated with an equal volume of pooled human serum. The mixture was incubated at 37 °C for 1 hour (keeping the zymosan in suspension). The opsonized zymosan was washed twice in PBS by centrifugation (300 g, 2 min), resuspended in PBS at 10 mg/mL and stored at −80 °C.

### Neutrophil uptake of NRT-cFLFLF. Flow cytometry analysis

Mice were intratracheal administered 50 μg LPS 24 hours prior the analysis. Later, the lung was thawed and excissed on R0 medium (*β-mercaptoethanol*, *Na-Piruvate*, *Glutamine*, *non-essential aminoacids and antibiiotics*), and exposed to enzimatic degradation being incubated for 30 minutes at 37 °C. Reaction was stopped with R10 medium (*RO medium supplemented with 10% FBS*) in ice. Homogenates were filtered and centrifuged 1700 rpm for 5 minutes.

Samples were processed into single cell suspension by adding RBC lysis buffer for 3 minutes RT. The reaction were stopped with R10 medium. Centrifuge at 1700 rpm for 5 minutes at 4 °C. Pellet was resuspended in FACS buffer.

A total of 100 ul of each homogenated was used for neutrophil quantification and fluorophore-conjugated (Alexa 488) ^68^Ga-NRT-cFLFLF neutrophil uptake. Samples were centrigue 1700 rpm for 5 5 minutes at 4 °C. Fc Block suspension were added to the pellet (1:200) and incubated for 15 minutes at 4 °C. Samples were washed and added antibody cocktail for 20 minutes incubation at 4 °C. To determine the cellular composition and differenciate neutrophil population, cells were stained with fluorochrome-labeled antibodies directed against CD45, Ly6G and CD11b (1:200, BD Bioscience).

Samples were washed twice with FACS buffer and final suspension was analysed using the FACSCanto™ II system (BD Biosciences). Samples were analysed with BD FACSDivaTM. Percentage of neutrophils in the lungs and fluorophore-conjugated ^68^Ga-NRT-cFLFLF neutrophil uptake were analysed using FlowJo Software (v10).

### Histolological analysis

At 24 hours after administration of LPS, mice were killed and the lungs were fixed by pefusing the animals with formalin and incubated in 10% formalin for 24 hours. Tissue was dehydrated and embedded in paraffin until sectioning. Lung sections were stained with Hematoloxilin & Eosin and Perl´s Prussian Blue. Images were processed and digitalised withNIS-Elements 3.22.11 acquisition software.

### *In vivo* cell depletion

For depletion of blood neutrophils, 50 μg of anti-Ly6G antibody (1A8 clone; BioXCell; West Lebanon, NH) was injected intraperitoneally for 2 consecutive days resulting in >90% reduction in blood neutrophil counts (*P* < 0.001); the levels of lymphocytes and monocytes in blood, or macrophages in BM were not affected by this treatment (not shown). For macrophage depletion, 100–150 μl of clodronate-loaded liposomes were intravenously injected per mouse one day prior to the experiments.

### Data availability

The authors declare that all data supporting the findings of this study are available within the paper and its Supplementary Information. Raw acquired PET/CT data can be made available upon reasonable request, with permission of the Fundación Centro Nacional de Investigaciones Cardiovasculares, Madrid, Spain.

## Electronic supplementary material

Supporting info
