# Supplementary material for: In vivo imaging of lung inflammation with neutrophil-specific 68Ga nano-radiotracer
Source: Sci Rep. 2017 Oct 16;7:13242. doi: 10.1038/s41598-017-12829-y (PMC5643527; doi:10.1038/s41598-017-12829-y)
Supplement: Supplementary file 1 — Supporting info [file 41598_2017_12829_MOESM1_ESM.doc]

***In vivo* imaging of lung inflammation with neutrophil-specific ^68^Ga nano-radiotracer**

*Juan Pellico^1^, Ana V. Lechuga-Vieco^1^, Elena Almarza^2,3^, Andrés Hidalgo^1,4^, Cristina Mesa-Nuñez ^2,3^, Irene Fernández-Barahona^1^, Juan A. Quintana^1^, Juan Bueren^2,3^, Jose A. Enríquez,^1^ Jesús Ruiz-Cabello^1,5^, Fernando Herranz^1^**

^1^ Centro Nacional de Investigaciones Cardiovasculares Carlos III (CNIC) and Centro de Investigación Biomédica en Red de Enfermedades Respiratorias (CIBERES). C/ Melchor Fernández-Almagro 3. 28029 Madrid. Spain.

^2^ Division of Hematopoietic Innovative Therapies, Centro de Investigaciones Energéticas Medioambientales y Tecnológicas / Centro de Investigación Biomédica en Red de Enfermedades Raras, 28040 Madrid, Spain.

^3^ Instituto de Investigación Sanitaria Fundación Jiménez Díaz (CIEMAT/CIBERER/IIS-FJD), 28040 Madrid, Spain.

^4^Institute for Cardiovascular Prevention, Ludwig-Maximilians-University 80336 Munich, Germany

^5^ Universidad Complutense de Madrid. 28040 Madrid. Spain

Supplementary information


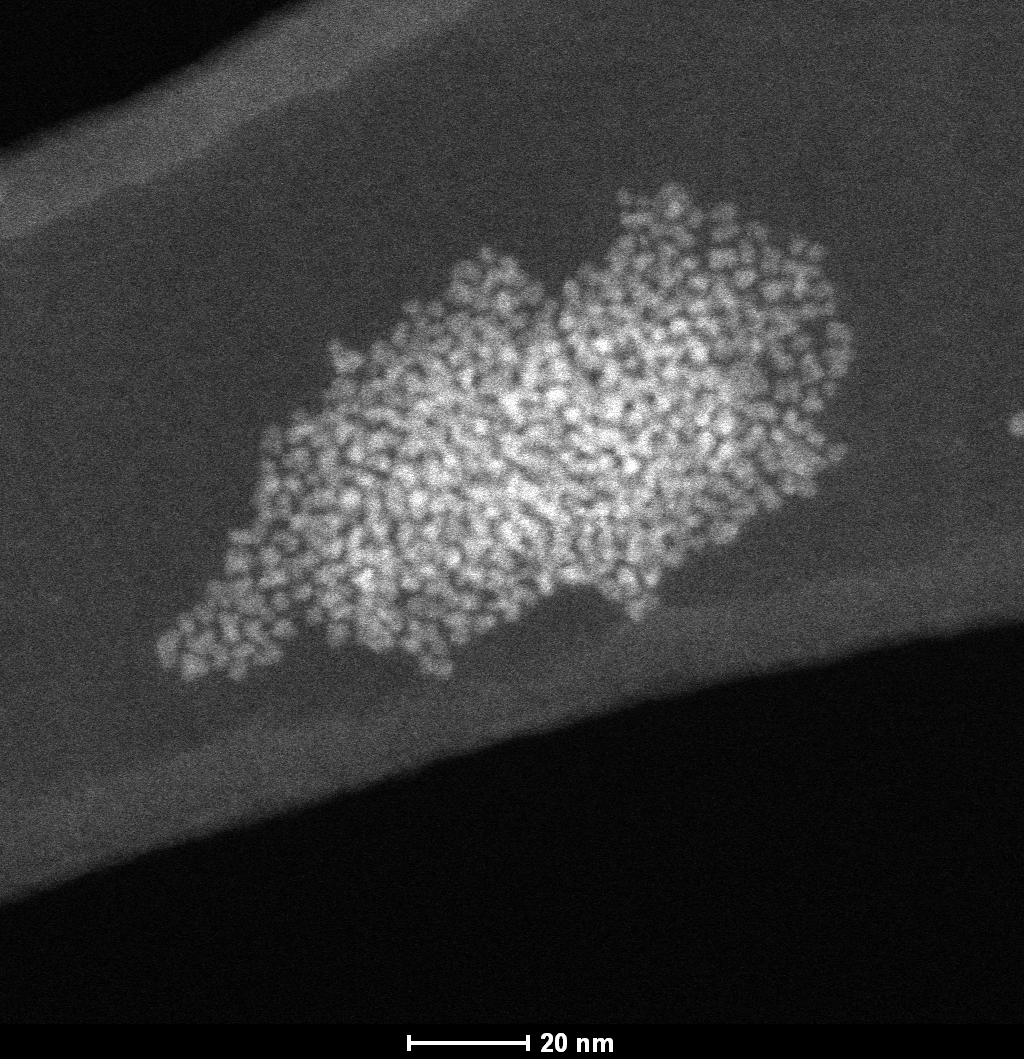


**Figure S1.** Representative electron microscopy image (STEM-HAADF) for ^68^Ga-NRT.

**Figure S2.** Thremogravimetric curve for ^68^Ga-NRT.

**Figure S3.** **a** Percentage of neutrophils in the lungs in control mice and mice with instilled LPS (****, *P* < 0.001 by two-tailed *t*-test; error bars indicate s.d., N = 4). Hematoxylin and eosin staining of lung sections from **b** a healthy mouse an LPS-instilled mouse and **c** a LPS-instilled mouse (scale bars are 100 μm).


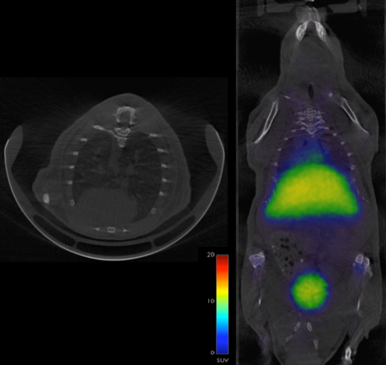


**Figure S4**. Representative PET/CT scan of LPS-induced lung inflammation in a mouse after injection of ^68^Ga-NRT.

**Figure S5.** Prussian blue staining of lung sections of mice injected with ^68^Ga-NRT-cFLFLF. **a** LPS-instilled mouse. **b** Healthy C57BL/6 mouse. **c** LPS-instilled mouse depleted of neutrophils.
